# Supplementary material for: The meritocracy trap: Early childhood education policies promote individual achievement far more than social cohesion
Source: PLoS One. 2025 Jul 2;20(7):e0326021. doi: 10.1371/journal.pone.0326021 (PMC12221063; doi:10.1371/journal.pone.0326021)
Supplement: Supporting Material 1 — (DOCX) [file pone.0326021.s001.docx]

Supplementary Materials for

**The meritocracy trap: Early childhood education policies promote individual achievement far more than social cohesion**

Katarzyna Bobrowicz, Pablo Gracia, Ziwen Teuber, Samuel Greiff

Corresponding author: [katarzyna.a.bobrowicz@gmail.com](mailto:katarzyna.a.bobrowicz@gmail.com)

S1 Table.

**Correlations among ratings of curricula from eleven randomly chosen countries submitted by independent raters (Spearman’s rho).**

| **Theme / Sub-theme** | **Indicator** | **Rater 1 test-retest** | **Rater 1 -Rater 2** | **Rater 1 – Rater 3** | **Decision** |
| --- | --- | --- | --- | --- | --- |
| Skills matter for lifetime success | Cognitive | .991*** | .964*** | .984*** | retain |
|  | Socio-emotional | .903*** | .828** | .832** | retain |
|  | Self-regulatory | .889*** | .883*** | .829** | retain |
|  | Physical | .937*** | .871*** | .816** | retain |
|  | Self-care | .941** | .828** | .886*** | retain |
|  | Artistic | .974*** | .840** | .838** | retain |
|  | Citizenship | .896*** | .884*** | .917*** | retain |
|  | Achievement | 1*** | .517 | .770** | retain |
|  | Strengths and weaknesses | .745** | .549 | .313 | drop |
| Individual agency and independence matter for lifetime success | Agency | .998*** | .989*** | - | retain |
|  | Initiative | .928*** | .823** | - | retain |
|  | Confidence | .824** | .716* | - | drop |
|  | Self-efficacy | .858*** | .601 | - | drop |
|  | Confidence and self-efficacy merged | .858*** | .759** | - | retain |
|  | Persistence | .216 | .165 | - | drop |
|  | Perseverance | .851*** | .996*** | - | drop |
|  | Persistence and perseverance merged | .833** | .827** | - | retain |
|  | Decision making | .939*** | .660* | - | retain |
|  | Effort | N/A | N/A | - | retain |
|  | Self as a source of success/failure | .861*** | .659* | - | retain |
|  | Independence | .982*** | .936*** | - | retain |
|  | Autonomy | .991*** | .881*** | - | retain |
|  | Self-direction | .956*** | .900*** | - | retain |
|  | Resilience | 1*** | .742** | - | retain |
| The uncontrollable matters for lifetime success | Career and income | 1*** | 1*** | 1*** | retain |
|  | Politics and economy | 1*** | N/A | N/A | drop |
|  | Risks and uncertainty | 1*** | 1*** | 1*** | retain |
|  | Lack of agency | N/A | N/A | N/A | retain |
| Individuals critically depend on teachers for lifetime success | Guidance | .990*** | .933*** | - | retain |
|  | Care | .967*** | .626* | - | retain |
|  | Collaboration | .871*** | .328 | - | drop |
|  | Teamwork | .875*** | .289 | - | drop |
|  | Collaboration and teamwork merged | .939*** | .512 | - | drop |
|  | Community | .867*** | .569 | - | drop |
|  | Common good | 1*** | .352 | - | drop |
|  | Solidarity | .885*** | .548 | - | drop |
|  | Inclusion | .973*** | .903*** | - | retain |
|  | Others as a source of success/failure | 0.744** | N/A | - | drop |
| Individuals critically depend on peers, family and the community for lifetime success | Guidance | .992*** | .574 | - | drop |
|  | Care | .516 | .191 | - | drop |
|  | Collaboration | .983*** | .581 | - | drop |
|  | Teamwork | .924*** | .833** | - | drop |
|  | Collaboration and teamwork merged | .981*** | .797** | - | retain |
|  | Community | .876*** | .679* | - | retain |
|  | Common good | 1*** | -.235 | - | drop |
|  | Solidarity | .997*** | 0.661* | - | retain |
|  | Inclusion | .978*** | 1*** | - | retain |
|  | Others as a source of success/failure | -.1 | N/A | - | drop |

The countries comprised Brazil, Bulgaria, Denmark, Finland, Iceland, Kenya, Malaysia, Taiwan, Sweden, and the United Kingdom (England and Northern Ireland). Indicators with at least one statistically insignificant score were dropped. Note: “N/A” stands for no occurrences of the indicator for one of the raters. * = p < .05; ** = p < .01; *** = p < .001.

S2 Table.

**Correlations among all indicators included in the analysis (Pearson’s r).**

|  | SOC-EMO | SELF-REG | PHYS | SELF-CARE | ART | CITIZEN | ACHIEV | CAREER | RISK | LACK | AGENCY | INIITIATIVE | CONFID | PERS | DECIS | EFFORT | SELFSUCCESS | INDEP | AUTON | SELF-DIR | RESIL | TEACH_GUIDE | TEACH_CARE | TEACH_INCL | OTHER_CARE | OTHER_COLTEAM | OTHER_COMMUNITY | OTHER_SOLID | OTHER_INCL |
| --- | --- | --- | --- | --- | --- | --- | --- | --- | --- | --- | --- | --- | --- | --- | --- | --- | --- | --- | --- | --- | --- | --- | --- | --- | --- | --- | --- | --- | --- |
| COG | -.05 | -.26 | .19 | .025 | .3 | .073 | -.093 | .13 | .099 | N/A | -.2 | -.18 | -.004 | .12 | -.23 | .09 | -.1 | -.24 | -.32 | -.33 | .09 | -.38 | -.25 | -.17 | -.22 | -.14 | .12 | -.041 | -.052 |
| SOC-EMO |  | .6 | -.12 | -.004 | .054 | .23 | -.068 | -.008 | -.03 | N/A | -.48 | -.28 | -.03 | -.11 | -.18 | -.1 | -.11 | -.43 | -.43 | -.38 | .027 | -.088 | -.065 | -.083 | .18 | -.16 | -.095 | -.096 | -.14 |
| SELF-REG |  |  | -.14 | .054 | -.12 | .053 | .058 | -.071 | -.082 | N/A | -.44 | -.09 | -.053 | -.043 | -.009 | -.032 | -.006 | -.26 | -.28 | -.18 | -.096 | -.043 | -.071 | -.1 | .28 | -.024 | -.015 | -.056 | -.11 |
| PHYS |  |  |  | .29 | .2 | .11 | -.15 | -.087 | -.096 | N/A | -.05 | -.27 | -.32 | .25 | -.23 | .056 | .083 | .078 | .066 | -.14 | -.2 | -.23 | -.14 | -.075 | -.13 | -.14 | -.27 | -.19 | -.13 |
| SELFCARE |  |  |  |  | .31 | .14 | -.039 | .24 | .2 | N/A | .03 | -.18 | -.12 | -.11 | -.19 | -.038 | -.14 | -.08 | -.11 | -.13 | -.1 | -.18 | -.075 | .056 | -.072 | -.14 | -.11 | .07 | .12 |
| ART |  |  |  |  |  | .14 | -.11 | -.036 | -.05 | N/A | -.17 | -.21 | -.024 | -.067 | -.11 | .05 | -.1 | -.31 | -.28 | -.26 | -.091 | -.11 | -.09 | .079 | -.15 | -.053 | -.052 | .085 | .15 |
| CITIZEN |  |  |  |  |  |  | -.042 | .13 | .099 | N/A | -.14 | .-.19 | -.013 | -.079 | -.12 | -.072 | -.098 | -.36 | -.36 | -.28 | -.026 | -.062 | -.018 | -.047 | -.093 | -.13 | -.1 | .015 | -.092 |
| ACHIEV |  |  |  |  |  |  |  | -.028 | -.038 | N/A | -.003 | .19 | -.051 | .037 | .25 | .037 | -.007 | -.1 | -.036 | .045 | -.06 | .043 | -.004 | -.033 | -.002 | .3 | .39 | .22 | -.0003 |
| CAREER |  |  |  |  |  |  |  |  | .9 | N/A | .069 | .12 | -.055 | -.036 | -.082 | -.031 | .06 | -.11 | -.094 | -.074 | -.024 | -.059 | -.018 | -.035 | -.031 | -.073 | -.064 | -.036 | -.049 |
| RISK |  |  |  |  |  |  |  |  |  | N/A | .073 | .085 | -.03 | -.04 | -056 | -04 | .071 | -.084 | -.067 | -.055 | -.013 | .021 | -.012 | .003 | -.045 | -.1 | -.072 | -.049 | -.055 |
| LACK |  |  |  |  |  |  |  |  |  |  | N/A | N/A | N/A | N/A | N/A | N/A | N/A | N/A | N/A | N/A | N/A | N/A | N/A | N/A | N/A | N/A | N/A | N/A | N/A |
| AGENCY |  |  |  |  |  |  |  |  |  |  |  | .44 | .055 | -.16 | .32 | .053 | -.096 | .34 | .36 | .33 | .02 | -.095 | -.23 | .26 | -.18 | .027 | -.096 | .009 | .15 |
| INITIATIVE |  |  |  |  |  |  |  |  |  |  |  |  | .14 | .032 | .51 | -.054 | .004 | .19 | .15 | .19 | .14 | -.12 | -.12 | .16 | .15 | .073 | -.039 | -.13 | -.069 |
| CONFID |  |  |  |  |  |  |  |  |  |  |  |  |  | .069 | .063 | -.035 | .03 | -.13 | -.14 | -.12 | .73 | .033 | -.031 | -.0007 | -.1 | .008 | -.06 | -.078 | .074 |
| PERS |  |  |  |  |  |  |  |  |  |  |  |  |  |  | -.045 | -.031 | .52 | -.2 | -.21 | -.15 | -.046 | -.049 | -.034 | -.063 | -.059 | .11 | .096 | -.075 | .17 |
| DECIS |  |  |  |  |  |  |  |  |  |  |  |  |  |  |  | -.007 | -.045 | -.055 | -.015 | .22 | -.11 | .11 | -.063 | .36 | -.14 | .22 | .002 | -.085 | .048 |
| EFFORT |  |  |  |  |  |  |  |  |  |  |  |  |  |  |  |  | .33 | .037 | -.03 | -.077 | -.062 | -.081 | -.026 | --081 | -.029 | .071 | -.014 | -.063 | .005 |
| SELF SUCCESS |  |  |  |  |  |  |  |  |  |  |  |  |  |  |  |  |  | -.029 | -.038 | .007 | -.1 | .0004 | -.066 | -.07 | .22 | -.089 | -.096 | -.11 | -.015 |
| INDEP |  |  |  |  |  |  |  |  |  |  |  |  |  |  |  |  |  |  | .92 | .57 | -.08 | -.25 | -.17 | -.23 | -.17 | .057 | -.15 | -.15 | -.2 |
| AUTON |  |  |  |  |  |  |  |  |  |  |  |  |  |  |  |  |  |  |  | .68 | -.078 | -.19 | -.17 | -.2 | -.17 | .11 | -.13 | -.11 | -.17 |
| SELF-DIR |  |  |  |  |  |  |  |  |  |  |  |  |  |  |  |  |  |  |  |  | -.19 | -.12 | -.13 | -.15 | -.11 | .23 | .018 | .022 | -.045 |
| RESIL |  |  |  |  |  |  |  |  |  |  |  |  |  |  |  |  |  |  |  |  |  | -.013 | -.035 | -.041 | -.06 | -.071 | -.089 | -.064 | -.089 |
| TEACH_GUID |  |  |  |  |  |  |  |  |  |  |  |  |  |  |  |  |  |  |  |  |  |  | .49 | .29 | .2 | -.14 | -.027 | .004 | .088 |
| TEACH_CARE |  |  |  |  |  |  |  |  |  |  |  |  |  |  |  |  |  |  |  |  |  |  |  | -.028 | -.022 | -.089 | -.046 | -.042 | -.042 |
| TEACH_INCL |  |  |  |  |  |  |  |  |  |  |  |  |  |  |  |  |  |  |  |  |  |  |  |  | -.048 | -.11 | -.005 | .29 | .49 |
| OTHER_CARE |  |  |  |  |  |  |  |  |  |  |  |  |  |  |  |  |  |  |  |  |  |  |  |  |  | -.006 | .087 | .14 | .011 |
| OTHER_COLTEAM |  |  |  |  |  |  |  |  |  |  |  |  |  |  |  |  |  |  |  |  |  |  |  |  |  |  | .53 | .46 | .23 |
| OTHER_COMMUN |  |  |  |  |  |  |  |  |  |  |  |  |  |  |  |  |  |  |  |  |  |  |  |  |  |  |  | .5 | .29 |
| OTHER_SOLID |  |  |  |  |  |  |  |  |  |  |  |  |  |  |  |  |  |  |  |  |  |  |  |  |  |  |  |  | .3 |

Five correlations greater than or equal to 0.6 occurred, between (1) socioemotional skills and self-regulatory skills, (2) career-income and risk-uncertainty, (3) confidence/self-efficacy and resilience, (4) independence and autonomy, and (5) autonomy and self-direction.

S3 Table. Overview of the dropped codes.

| **Theme/Sub-theme** | **Indicator** | **Definition** |
| --- | --- | --- |
| Skills matter for lifetime success | Strengths and weaknesses | Explicitly mentioned abilities, or strengths and/or weaknesses of the individual. |
| The uncontrollable matters for lifetime success | Politics and economy | Explicit mentions of politics and economy alongside one another. |
| Individuals critically depend on teachers for lifetime success | Collaboration and teamwork | Child’s engagement in collaboration or cooperation with others, typically to achieve a common goal. Also child’s active involvement in groups or teams. |
|  | Community | Child’s reliance on and knowledge of others in the community, their roles, and/or contribution to the community life. |
|  | Common good | Child’s understanding of interdependence with other members of the group, community or society in securing the values, resources and other goods shared by the members. |
|  | Solidarity | Child’s readiness to help others in need and awareness of dependence on others for such help. |
|  | Others as a source of success/failure | Others’ influence on/control over the outcomes of the child’s behaviors. |
| Individuals critically depend on peers, family and the community for lifetime success | Guidance | Child’s behavior through which s/he seeks guidance, support, and modelling, OR adult behavior through which guidance, support and modelling are offered to the child. |
|  | Care | Child’s behavior through which s/he seeks physical and/or emotional care, OR adult behavior through which physical and/or emotional care are offered to the child. |
|  | Common good | Child’s understanding of interdependence with other members of the group, community, or society in securing the values, resources and other goods shared by the members. |
|  | Others as a source of success/failure | Others’ influence on/control over the outcomes of the child’s behaviors. |

S1 File. (separate file). An overview of a composition of policy decrees and ECE curricula across the OECD, the EU, and the fifty-four countries included in the study.
