# Supplementary material for: The meritocracy trap: Early childhood education policies promote individual achievement far more than social cohesion
Source: PLoS One. 2025 Jul 2;20(7):e0326021. doi: 10.1371/journal.pone.0326021 (PMC12221063; doi:10.1371/journal.pone.0326021)
Supplement: Attachment — Submitted filename: Confidential Review_PONE-D-24-28449.pdf [file pone.0326021.s003.pdf]

Confidential Review
Manuscript ID: PONE-D-24-28449
PLOS ONE

Dear Authors,

Thank you for the opportunity to review this manuscript. I commend your efforts in exploring whether reformed Early Childhood Education (ECE) may lay the foundation for an economic meritocratic mindset across **fifty-four countries**. The results of your thematic analyses are both intriguing and potentially impactful in the fields of ECE and related educational disciplines, particularly in the context of developing policies and reforms that consider a meritocratic mindset. Below, I provide detailed comments and suggestions that I believe will enhance the clarity, structure, and overall contribution of your work:

1. The introduction effectively builds the arguments necessary to examine the hypotheses. However, while the authors state that the role of meritocratic ideologies within the context of ECE has been overlooked in previous research, I did not find supporting evidence for this claim. To strengthen the manuscript, I suggest either presenting related arguments or citing earlier studies that highlight this gap in the literature. Additionally, it is important to consider that readers are interested in understanding the rationale for conducting the research, not simply because it fills a gap in the literature. If this research area is indeed underexplored, it would be more compelling to build arguments around the significance and potential impact of studying this topic, rather than merely stating that it is a gap. There are many unexplored phenomena in the literature, but the key question is: why is it important to investigate this particular phenomenon?

2. The authors state, "The hypothesis may be rejected if education stakeholders indicate that (1) other factors, such as the current market value of one's skills and uncontrollable external events, can critically influence one's lifetime success" (p. 6). In research, it is important to provide supporting arguments for these factors. I noticed that the study does not include a Literature Review section, which could be a suitable place to present these factors in detail. While the current market value of skills and uncontrollable external events are mentioned, the manuscript does not provide a clear flow of ideas to guide readers in recognizing the significance of these potential factors. Including a Literature Review or integrating more supporting arguments could improve the coherence and depth of the discussion.

3. I am not entirely certain whether *PLOS ONE* requires a section on Theoretical, Conceptual, and/or Analytical Frameworks. However, I strongly recommend integrating these frameworks into your manuscript, as their omission could undermine the credibility of the results. Specifically, it is challenging to accept the results presented in the study without a detailed explanation of its foundational elements, such as the Theoretical and Analytical Frameworks, especially given the use of thematic analysis. An Analytical Framework, for instance, would provide valuable insight into the origin of the trends, themes, and patterns identified by the authors. Including these frameworks is not only essential for the rigor of the research but also beneficial for other researchers who may wish to replicate the study in different contexts.

4. The authors state, "We defined uncontrollable external events as **society-wide political and economic contexts, the monetary value of different careers, and events where individual agency is limited or none**" (p. 7). While I can accept these events as

variables, I would like to see examples of how such events are incorporated into the research questions (hypotheses). The absence of a Literature Review makes it challenging to justify their inclusion in the study. It is easy to identify variables that seem relevant and interesting, but justifying why these particular variables should be part of the phenomenon under investigation requires robust arguments (rationale). Consider this question: why should these specific variables (as highlighted above) be selected over others? I am not trying to make this process difficult for the authors, but as a reader, I would appreciate seeing how these variables are integrated into the research. Please reflect on this question when revisiting the hypotheses.

5. The Analytical Framework guides the selection of the analysis method, and in this research, the authors employed thematic analysis. However, it is unclear how the authors conducted this thematic analysis. Specifically, how were the themes in Table 2 identified? What was the rationale for retaining these particular themes? Additionally, how many coders were involved in the analysis, and how were discrepancies between the coders' results resolved? These important details should be clearly outlined in a Method section, which is currently missing from the manuscript. Including this information would enhance the transparency and reliability of the research process, thereby strengthening the fidelity of the study's findings. Furthermore, since the study leans towards a qualitative approach, it is important to explain how triangulation was addressed.

6. The authors state, "Themes and indicators of a meritocratic mindset were identified based on previous literature and close, iterative reading of the data (for details consult Materials and Methods)" (p. 14). However, I could not locate a Materials and Methods section. Toward the end of the article, I found supplementary materials, which I assume is what the authors referred to. My suggestion is to incorporate the methodological analysis into a dedicated Method section within the manuscript. Additionally, the rationale for selecting Spearman's rho to find correlations among ratings of curricula should be clearly justified. Why was this method chosen instead of inter-rater correlations, given that the raters conducted the thematic analysis? If the authors followed a different analytical process, it should be clearly explained in the manuscript. For instance, it is unclear how the data in the Excel were generated, as there is no description or explanation provided. The manuscript primarily mentions that thematic analysis was conducted, but the details of this process need to be thoroughly articulated.

7. The authors state, "The analysis confirmed that experts and policymakers widely recognize the importance of universal and equal access to ECE" (p. 14). However, it is unclear which analysis this statement refers to. I read through the manuscript multiple times but could not find data or findings supporting this claim in the manuscript. It would be helpful to clarify the specific analysis that led to this conclusion and ensure that the supporting data or analysis is clearly presented in the manuscript.

8. In the Results section, the primary focus should be on addressing the hypotheses. Are they rejected or accepted based on the analyses and results? I recommend restating each of the two hypotheses when reporting the results. This approach will help the audience stay clear about what the authors are reporting and ensure that the connection between the results and the hypotheses is evident.

I have reviewed the Results and Discussion sections, and while they present interesting findings, the clarity and persuasiveness of these sections are compromised by the lack of a smooth flow of ideas and presentation throughout the manuscript. This issue is particularly evident in the

absence or lack of clarity in the Theoretical, Conceptual, and/or Analytical Frameworks, Literature Review, and Methods sections. Without a strong foundation in these areas, it is challenging for the Results and Discussion sections to receive the attention and consideration they deserve. I encourage the authors to rework include these foundational sections first, as this will allow for a more coherent and impactful presentation of the Results and Discussion. Furthermore, I have raised a few concerns in my previous comments regarding the findings and claims reported in the Results section, which I hope will guide the revision process.

The suggestions provided are intended to support further refinement of your manuscript, offering an external perspective that I hope you will find valuable. I appreciate the opportunity to contribute to your work and look forward to seeing a revised version should you choose to resubmit to *PLOS ONE*.

Best wishes,
Reviewer
