# Supplementary material for: The meritocracy trap: Early childhood education policies promote individual achievement far more than social cohesion
Source: PLoS One. 2025 Jul 2;20(7):e0326021. doi: 10.1371/journal.pone.0326021 (PMC12221063; doi:10.1371/journal.pone.0326021)
Supplement: Attachment — Submitted filename: Response to reviewers.docx [file pone.0326021.s005.docx]

Dear Editorial team and Reviewer 1,

We are glad that the Editorial team and the anonymous reviewer see merits in our paper considered for publication at *PLOS ONE*. Reviewer 1 has provided excellent suggestions and comments that we have carefully considered to improve our manuscript. Below, we provide a specific answer to each point raised by Reviewer 1. We have carefully answered each point and accordingly made important improvements to the and clarified any issues in this response letter. To ease transparency and clarity in the reviewing process, the changes introduced to the manuscript are now highlighted in yellow throughout the manuscript.

In our own reading of the manuscript, we have noticed that the analysis involved fifty-three, not fifty-four countries, as the commercial and the governmental curricula from the United States of America were considered as coming from two different countries. We have now amended this throughout the manuscript. Furthermore, we have amended Fig 2 to include data from France among the pie charts, which has been omitted in the original version of the figure. Finally, we have amended the title to better reflect the message of the manuscript and its relationship to a key source (“The Meritocracy Trap” by D. Markovits).

We are looking forward to further feedback from the Editorial team and the Reviewer in the hope that the revised version of the manuscript is meeting the publication standards of *PLOS ONE*.

Best regards,

The Authors

**Response to the Editor**

*1. Please ensure that your manuscript meets PLOS ONE's style requirements, including those for file naming. The PLOS ONE style templates can be found at*

*https://journals.plos.org/plosone/s/file?id=wjVg/PLOSOne_formatting_sample_main_body.pdf and*

[*https://journals.plos.org/plosone/s/file?id=ba62/PLOSOne_formatting_sample_title_authors_affiliations.pdf*](https://journals.plos.org/plosone/s/file?id=ba62/PLOSOne_formatting_sample_title_authors_affiliations.pdf)

In the revised submission, we have followed the PLOS ONE formatting guidelines and the templates for file naming.

**2. We note that Figure 1 in your submission contain [map/satellite] images which may be copyrighted. All PLOS content is published under the Creative Commons Attribution License (CC BY 4.0), which means that the manuscript, images, and Supporting Information files will be freely available online, and any third party is permitted to access, download, copy, distribute, and use these materials in any way, even commercially, with proper attribution. For these reasons, we cannot publish previously copyrighted maps or satellite images created using proprietary data, such as Google software (Google Maps, Street View, and Earth). For more information, see our copyright guidelines:** [**http://journals.plos.org/plosone/s/licenses-and-copyright**](http://journals.plos.org/plosone/s/licenses-and-copyright)**.**

Many thanks for drawing our attention to this matter. The map was generated through a standard “ggplot” function relying on the following packages in R: “ggplot2”, “ggpubr”, “rnaturalearth”, “rnaturalearthdata”, “cowplot”, “googleaway”, “ggrepel”, “ggspatial”, “libwgeom”, “sf”. Using R for this purpose has now been clarified in legend of Fig 1 (page 41). We have not cited all these packages to save space in the current revised version.

The following code was used to generate Fig 1:

“require(ggplot2)

require("rnaturalearth")

require("rnaturalearthdata")

require("cowplot")

require("googleaway")

require("ggrepel")

require("ggspatial")

require("libwgeom")

require("sf")

world <- ne_countries(scale = "medium", returnclass = "sf")

class(world)

ggplot(data = world) +

geom_sf()

ggplot(data = world) +

geom_sf(color = "darkgray", fill = "white")

map<-map_data("world")

require(dplyr)

\# Set colors

map <- mutate(map, fill = ifelse(region %in% c("Ethiopia",

"Kenya",

"South Africa",

"Tanzania",

"China",

"Hong Kong",

"India",

"Japan",

"Malaysia",

"Pakistan",

"Philippines",

"South Korea",

"Singapore",

"Taiwan",

"Australia",

"New Zealand",

"Albania",

"Austria",

"Belgium",

"Bulgaria",

"Croatia",

"Czechia",

"Denmark",

"Estonia",

"Finland",

"France",

"Germany",

"Iceland",

"Ireland",

"Italy",

"Latvia",

"Lithuania",

"Luxembourg",

"Norway",

"Poland",

"Portugal",

"Romania",

"Slovakia",

"Slovenia",

"Spain",

"Sweden",

"Netherlands",

"UK",

"Jordan",

"Israel",

"Saudi Arabia",

"Turkey",

"Canada",

"Mexico",

"USA",

"Argentina",

"Brazil",

"Chile"), "navy", "white"))

\# Use scale_fiil_identity to set correct colors

ggplot(map, aes(long, lat, fill = fill, group=group)) +

geom_polygon(colour="lightgray")+

scale_fill_identity()+

theme(axis.title = element_blank(),

axis.ticks = element_blank(),

axis.text =element_blank())”

**Response to the Reviewer**

*Thank you for the opportunity to review this manuscript. I commend your eﬀorts in exploring whether reformed Early Childhood Education (ECE) may lay the foundation for an economic meritocratic mindset across ﬁfty-four countries. The results of your thematic analyses are both intriguing and potentially impactful in the ﬁelds of ECE and related educational disciplines, particularly in the context of developing policies and reforms that consider a meritocratic mindset. Below, I provide detailed comments and suggestions that I believe will enhance the clarity, structure, and overall contribution of your work:*

We want to thank Reviewer 1 for providing such detailed and constructive review which has been instrumental to revising and improving our manuscript.

1. *The introduction eﬀectively builds the arguments necessary to examine the hypotheses. However, while the authors state that the role of meritocratic ideologies within the context of ECE has been overlooked in previous research, I did not ﬁnd supporting evidence for this claim. To strengthen the manuscript, I suggest either presenting related arguments or citing earlier studies that highlight this gap in the literature. Additionally, it is important to consider that readers are interested in understanding the rationale for conducting the research, not simply because it ﬁlls a gap in the literature. If this research area is indeed underexplored, it would be more compelling to build arguments around the signiﬁcance and potential impact of studying this topic, rather than merely stating that it is a gap. There are many unexplored phenomena in the literature, but the key question is: why is it important to investigate this particular phenomenon?*

We thank Reviewer 1 for this comment. We fully agree with Reviewer 1 that the previous version of the paper required more direct engagement with previous scholarship to motivate the topic and our study in the context of existing literature. In the introduction of the new version of the paper we have now:

a) contextualized the present study in relation to previous research on neoliberalism, the Human Capital Theory, and meritocracy in educational policy in ECE and beyond (pages 5-6). We have highlighted the gaps in the literature, while also situating previous literature and previous arguments and research more in depth (pages 4 and 6).

b) stressed more directly the motivation of this topic from the beginning in the introduction of the manuscript (page 3).

Finally, in line with the comments from Reviewer 1, we have also revised the abstract to communicate more effectively the motivation, objectives, methodology, results and overall conclusion of our study (page 2).

2. *The authors state, “The hypothesis may be rejected if education stakeholders indicate that other factors, such as the current market value of one’s skills and uncontrollable external events, can critically inﬂuence one’s lifetime success” (p. 6). In research, it is important to provide supporting arguments for these factors. I noticed that the study does not include a Literature Review section, which could be a suitable place to present these factors in detail. While the current market value of skills and uncontrollable external events are mentioned, the manuscript does not provide a clear ﬂow of ideas to guide readers in recognizing the signiﬁcance of these potential factors. Including a Literature Review or integrating more supporting arguments could improve the coherence and depth of the discussion.*

We agree that the background and the literature review were underdeveloped in the original version of the manuscript. In the revised version, we have (1) added a Literature Review section in the Background, (2) revised and improved the theoretical framework building on previous research in this area more explicitly, and (3) clarified more thoroughly what is meant by the current market value of skills and uncontrollable external events (pages 4 and 7).

3. *I am not entirely certain whether PLOS ONE requires a section on Theoretical, Conceptual, and/or Analytical Frameworks. However, I strongly recommend integrating these frameworks into your manuscript, as their omission could undermine the credibility of the results. Speciﬁcally, it is challenging to accept the results presented in the study without a detailed explanation of its foundational elements, such as the Theoretical and Analytical Frameworks, especially given the use of thematic analysis. An Analytical Framework, for instance, would provide valuable insight into the origin of the trends, themes, and patterns identiﬁed by the authors. Including these frameworks is not only essential for the rigor of the research but also beneﬁcial for other researchers who may wish to replicate the study in diﬀerent contexts.*

We fully agree with Reviewer 1 in that adding an Analytical Framework provides valuable insight into the origin of the trends, themes, and patterns identiﬁed”. As mentioned in point 2, we have extensively added a new section for these purposes and then improved our Background, in terms of literature review and theoretical expectations (pages 5-6). Our methodological approach is spelled out and inserted right after the Background in the revised manuscript (pages 8-12).

4. *The authors state, “We deﬁned uncontrollable external events as society-wide political and economic contexts, the monetary value of diﬀerent careers, and events where individual agency is limited or none” (p. 7). While I can accept these events as variables, I would like to see examples of how such events are incorporated into the research questions (hypotheses). The absence of a Literature Review makes it challenging to justify their inclusion in the study. It is easy to identify variables that seem relevant and interesting, but justifying why these particular variables should be part of the phenomenon under investigation requires robust arguments (rationale). Consider this question: why should these speciﬁc variables (as highlighted above) be selected over others? I am not trying to make this process diﬀicult for the authors, but as a reader, I would appreciate seeing how these variables are integrated into the research. Please reﬂect on this question when revisiting the hypotheses.*

We fully agree with Reviewer 1 in that justifying this conceptualization based on previous literature and on an argument that derives from it is important for the study. Therefore, the background has been changed globally following these recommendations from Reviewer 1, including an explanation of “uncontrollable factors” in the Introduction (page 4).

5. *The Analytical Framework guides the selection of the analysis method, and in this research, the authors employed thematic analysis. However, it is unclear how the authors conducted this thematic analysis. Speciﬁcally, how were the themes in Table 2 identiﬁed? What was the rationale for retaining these particular themes? Additionally, how many coders were involved in the analysis, and how were discrepancies between the coders’ results resolved? These important details should be clearly outlined in a Method section, which is currently missing from the manuscript. Including this information would enhance the transparency and reliability of the research process, thereby strengthening the ﬁdelity of the study's ﬁndings. Furthermore, since the study leans towards a qualitative approach, it is important to explain how triangulation was addressed.*

We appreciate this remark, and we have striven to clarify the entire methodological process behind the analysis. In line with the Reviewer’s feedback and the PLOS One guidelines, we have now included a “Materials and methods” section between the Background and the Results. done it. Critically, in the revised manuscript, we have extensively revisited the method by accounting for the Reviewer’s questions on the flow of the thematic analysis (pages 9-11), the definition of the themes and the rationale behind them (page 10), and the combination of the qualitative and the quantitative analyses (page 12).

6. *The authors state, “Themes and indicators of a meritocratic mindset were identiﬁed based on previous literature and close, iterative reading of the data (for details consult Materials and Methods)” (p. 14). However, I could not locate a Materials and Methods section. Toward the end of the article, I found supplementary materials, which I assume is what the authors referred to. My suggestion is to incorporate the methodological analysis into a dedicated Method section within the manuscript. Additionally, the rationale for selecting Spearman’s rho to ﬁnd correlations among ratings of curricula should be clearly justiﬁed. Why was this method chosen instead of inter-rater correlations, given that the raters conducted the thematic analysis? If the authors followed a diﬀerent analytical process, it should be clearly explained in the manuscript. For instance, it is unclear how the data in the Excel were generated, as there is no description or explanation provided. The manuscript primarily mentions that thematic analysis was conducted, but the details of this process need to be thoroughly articulated.*

This is an important question. In the first (older) submission, the order of sections was confusing for the format of PLOS One. We have now adjusted it in line with the journal guidelines and the Reviewer’s comments. First, as explained above, the Materials and methods section is now located between the Background and Results sections. Second, the supplemental tables have been added within the same document of the paper at the end, to ensure the Reviewer’s access. Tables and figures are clearly cited in this regard. Third, in the revised manuscript, we explain into detail why (i) we used Spearman’s rho to ﬁnd correlations among ratings of curricula and (ii) how we use inter-rater correlations in the analyses (pages 10-11). Finally, we have now verified that the way we handled the analyses is clearly explained in the Materials and methods section, including information on the supporting dataset (S1 File) analyzed in the study.

7. *The authors state, “The analysis conﬁrmed that experts and policymakers widely recognize the importance of universal and equal access to ECE” (p. 14). However, it is unclear which analysis this statement refers to. I read through the manuscript multiple times but could not ﬁnd data or ﬁndings supporting this claim in the manuscript. It would be helpful to clarify the speciﬁc analysis that led to this conclusion and ensure that the supporting data or analysis is clearly presented in the manuscript.*

This remark refers to the reading of the OECD and the EU documents listed in Table 2, followed by the thematic analysis that quantified the frequency of occurrence for relevant indicators in these documents when addressing hypotheses H1 and H2. We have now clarified this statement by stating that “The close reading of the policymakers’ reports (see Table 2…” was carried out (page 12).

8. *In the Results section, the primary focus should be on addressing the hypotheses. Are they rejected or accepted based on the analyses and results? I recommend restating each of the two hypotheses when reporting the results. This approach will help the audience stay clear about what the authors are reporting and ensure that the connection between the results and the hypotheses is evident.*

To address this comment, we have revised the two hypotheses as Hypothesis H1 and H2. Thereafter, we have revised the structure of the Results in order to clearly indicate which sections pertain to each of the hypotheses. Two short paragraphs, relating the Results to the hypotheses have been introduced (H1: page 13, H2: page 15), and further addressed in the Discussion (pages 15-16).

*I have reviewed the Results and Discussion sections, and while they present interesting ﬁndings, the clarity and persuasiveness of these sections are compromised by the lack of a smooth ﬂow of ideas and presentation throughout the manuscript. This issue is particularly evident in the absence or lack of clarity in the Theoretical, Conceptual, and/or Analytical Frameworks, Literature Review, and Methods sections. Without a strong foundation in these areas, it is challenging for the Results and Discussion sections to receive the attention and consideration they deserve. I encourage the authors to rework include these foundational sections ﬁrst, as this will allow for a more coherent and impactful presentation of the Results and Discussion. Furthermore, I have raised a few concerns in my previous comments regarding the ﬁndings and claims reported in the Results section, which I hope will guide the revision process.*

We appreciate this feedback. This comment has guided us in the current revision round. We have revised all sections mentioned by the Reviewer, connecting the key elements of the manuscript and bringing them together at the end of the Discussion.

*The suggestions provided are intended to support further reﬁnement of your manuscript, oﬀering an external perspective that I hope you will ﬁnd valuable. I appreciate the opportunity to contribute to your work and look forward to seeing a revised version should you choose to resubmit to PLOS ONE.*

We thank Reviewer 1 for the constructive critique of the manuscript, as it has been extremely useful for improving our manuscript. We hope that the concerns raised by the Editorial team and Reviewer 1 have been sufficiently addressed in the revised manuscript, yet we remain open to further revisions.
