# Supplementary material for: The meritocracy trap: Early childhood education policies promote individual achievement far more than social cohesion
Source: PLoS One. 2025 Jul 2;20(7):e0326021. doi: 10.1371/journal.pone.0326021 (PMC12221063; doi:10.1371/journal.pone.0326021)
Supplement: Attachment — Submitted filename: Revision 2_Response to reviewers.docx [file pone.0326021.s006.docx]

Dear Editors and Reviewers,

Thank you very much for the opportunity to revise and resubmit the paper considered for publication in *PLOS One*.

We have carefully read and implemented the very relevant suggestions from Reviewer 2. Below we provide an answer to each comment raised by Reviewer 2 and reference to the action plan implemented in relation to these comments. We believe that our paper is now stronger and improved thanks to the feedback received through the hitherto revision rounds at *PLOS One*.

Best regards,

The Authors

**RESPONSE TO REVIEWER 2**

*Reviewer #2:*

*This is a very interesting paper, and the revisions are important and substantive. Although I was not a first-round reviewer, the authors have engaged substantively with the comments and it has made for a much improved paper. There are two real issues that I would suggest for minor revision.*

We are glad to hear that Reviewer 2 has found our paper very interesting and also values the extensive work and improvements made on the manuscript in the previous version of the paper.

*First, there is a seminal paper written by Carneiro & Heckman from 2003 [Carneiro, P. M., & Heckman, J. J. (2003). Human capital policy]. After this paper, Heckman used his significant power and advantage in the field of economics to explicitly promote ECE as "the best ROI." This not only turned ECE into an "economic investment in meritocracy" but also specifically set out via intermediary orgs, international NGOs and policy entrepreneurs to invest the world in ECE as prevention of bad outcomes like public dependency or crime (like public health) and the production of good outcomes (executive functioning, emotional regulation, and human capital more broadly). First, this paper should definitely be included in your lit review, as should many of those that follow & cite this paper. It also may need to cite some of the foci of the programs that were included in Heckman's original analyses and their foci—Head Start, for example, had a very large focus on citizenship, community advancement, and equity. So here, it's worth noting what the human capital focus by Carneiro & Heckman lifts to the top & leaves behind.*

We thank Reviewer 2 for this suggestion. We have now accordingly included the Carneiro and Heckman (2003) study in our literature review, along with a discussion of its outsized influence in shaping ECE as an economic investment, particularly through Heckman’s broader advocacy work. We also reference subsequent work that amplified this economic framing, including Bruner (2004), to highlight how these ideas circulated via policy networks and intermediaries. In addition, we have referred to programs like Head Start—emphasizing citizenship, community, and equity—in relation to this literature.

*However, the inclusion of this paper and its role in the proliferation of the meritocratic promises of ECE present a few challenges & opportunities that this history presents for your findings as currently written:*

*1) The spread of meritocratic ideas is currently unembodied in your paper. Yet, any study of diffusion & institutionalization would note that intermediary orgs, bodies of evidence, and institutional entrepreneurs are typically pushing particular ways of seeing and using evidence to achieve policy ends. Here's an example of this that followed Heckman's article within the year following its publication [Bruner, C. (2004). Many happy returns: Three economic models that make the case for school readiness.].*

We thank Reviewer 2 for drawing our attention to this matter. We have responded by explicitly incorporating the role of intermediary organizations, institutional entrepreneurs, and policy networks into our discussion of how ECE rationales are diffused and institutionalized. In particular, we refer to Bruner (2004) as a clear example of how economic models were quickly mobilized to support early learning investments. This allows us to better contextualize how the meritocratic framing of ECE has been promoted and sustained beyond the academic sphere.

*2) If this is the case, then it's possible that the values written about are simply the articulations that neoliberal governments use to justify the policy of ECE... e.g., governments won't make the case for ECE on these other means and the only ones that do are actually demonstrating a measure of "looser ties" with the intermediary orgs that spread ECE policy. So, your measures might in fact demonstrate the level of influence of this kind of tight ties among the neoliberal countries but not actually reflect the reality of ECE programs or values on the ground. This is especially likely given that ECE & almost all K-3 education is notoriously localized and quite resistant to efforts to standardize due to a lack of common training & certification of teachers & low pay in many countries. So, you would want to clarify at what level you think the ideology is reflected (I don't think you're saying it's for sure happening on the ground, but you'll want to be very clear about your language to designate that this is the way countries are justifying their investments & priorities to external audiences).*

We appreciate this comment and we agree with Reviewer 2. In the manuscript, we focus on what states, through their ECE policies, are aiming to promote in ECE facilities, acknowledging that such policies may cause resistance among teachers across countries, which may, however, dissipate with increased state support for implementing the policies. Therefore, in the revised version, we explicitly state that (1) educational stakeholders may hold diverse views, and (2) we propose that future studies should further investigate these dynamics across institutions and stakeholders.

*3) The opportunity here is that in the implications/discussion, there are a few missing that really matter for kids and families as well as the take-up of ECE: a) Marketing mismatches may not appeal to families based on their cultural values that is, countries may articulate a meritocratic rationale and it may not resonate [see this example: Patnaik, N. (2023). Teaching development: Critically evaluating ECE for economic growth in India. International Journal of Social Science & Economic Research, 8(08), 2390–2396.]; b) Mismatches between the promises made for ECE and people's experiences may support a backlash or divestment in countries where there is a large uptake (i.e., "my ECE program is low-quality, didn't help my child", etc.); c) The meritocratic promise doesn't only affect individual expectations, it creates a competitive market for ECE in places where it's privatized as families seek to "get ahead" via best ECE like best colleges; and d) ECE teachers who do focus on citizenship and/or social skills may experience pressure from families to "teach hard skills" for individual success (that is, parents as consumers may both believe the marketing & reinforce its expectation on their ECE providers). Fleshing out these real-world implications of your research may help strengthen the rationale for the work you've done & demonstrate that a nation's articulated rationale for investing in ECE may actually, in the long-run, determine its focus and content over time.*

We thank Reviewer 2 discussing these relevant implications. We have added a new part in the discussion to reflect on the broader real-world consequences of meritocratic ECE framing. This includes your point about potential mismatches between policy narratives and families’ cultural values (e.g., Patnaik, 2023), the risk of backlash if expectations are unmet, the emergence of competitive ECE markets in privatized settings, and the pressures placed on educators to conform to a more academic or skills-based approach. We believe these additions deepen our analysis of how policy discourse can shape family perceptions and educator practices, with real-world implications to strengthen the rationale of our paper in terms of the implication.

We have highlighted the added paragraph in the revised version of the manuscript.

*Best of luck as you continue to revise this paper. It's meaningful and important work, and I'd suggest that you follow-up with a paper on the proliferation of this model & its origins globally.*

We are very grateful for your encouraging words and your suggestion to pursue this line of inquiry further. We hope that Reviewer 2 is satisfied with the changes made to the manuscript.
